# Missed Opportunities in Geriatric Oncology Research

**DOI:** 10.1093/oncolo/oyad072

**Published:** 2023-03-20

**Authors:** Stuart M Lichtman

**Affiliations:** Wilmot Cancer Institute Geriatric Oncology Research Group, University of Rochester, Rochester, NY, USA

**Keywords:** geriatric oncology, medical oncology, clinical trials, data reporting, data analysis

## Abstract

The field of geriatric oncology has made significant progress in recent decades, but there are still missed opportunities in important areas of research. One issue is the underrepresentation of older patients, especially those aged 75 years and older, in clinical trials. This has resulted in a lack of high-quality data for the care of this population, and the American Society of Clinical Oncology has called for an increase in the evidence base for older patients with cancer. The second missed opportunity is the chance to gather important knowledge from older patients participating in clinical trials, such as medications, social support, insurance, and financial information. These data can be easily collected and incorporated into the trial design to enhance the information available to researchers and clinicians. The third missed opportunity is the chance to robustly analyze and report clinical trial data for the benefit of geriatric oncology research. Many trials only report a median age and range, which is a disservice to both the participants and the patients who will be treated based on the study results. To advance geriatric oncology research, the necessary data need to be collected, analyzed, and reported through appropriate representation of older patients, collection of essential information, and thorough analysis and communication of results. Clinical trial design needs to include geriatric baseline parameters, and Cancer Therapy Evaluation Program (CTEP) has modified its template to include these parameters.

Every patient on a clinical trial is a valuable resource. These patients submit to the rigors of a clinical trial in order not only to improve their own situations but also to contribute to advances in cancer care and research. At study completion, patients’ results are reported to help clinicians make important care decisions, particularly if the study is a success and a new therapy is approved.

Over the past 30 years, the field of geriatric oncology has made significant advancements but gaps remain in important areas of research. Three significant and interrelated missed opportunities are discussed here. First, older patients, particularly those aged 75 years and older, are consistently underrepresented in clinical trials.^1-6^

The second missed opportunity is the chance to gather important knowledge from older patients taking part in clinical trials. This applies to single-agent studies, randomized phase II trials, and, most importantly, pivotal randomized phase III studies; as well as to studies that address solid tumors or hematologic malignancies. Typical clinical trial designs, such as in Cancer Therapy Evaluation Program (CTEP) templates, require the listing of basic demographic information such as age, sex, and race, as well as commodities, laboratory studies, and pertinent past medical history. The recording of medications, social supports, insurance, or financial information is rarely required, and no attempt is made to evaluate functional status of the patient.^6-12^ Consequently, while clinicians rely on the results of these trials to help create treatment plans and feel obliged to use the dose and schedule results, the granular information needed to personalize treatment to individual patients does not exist. It is essential that this issue is addressed in studies of diseases that are inherently malignancies of older patients, such as prostate cancer and multiple myeloma.

The relatively simple intervention of including these additional data in clinical trial design would greatly enhance the information available to researchers and clinicians at very little cost or extra expenditure of time for the clinical staff. It has been amply demonstrated that self-administered geriatric assessment is accurate and cost and time effective.^13^ The collection of data regarding medications, social supports, insurance, and financial information can be easily incorporated into the process of collecting basic demographic information and past medical history. Office staff also can be trained to do the functional assessments, such as the “get-up-and-go” test and handgrip assessment. The simple act of watching the patient walk, evaluating his or her gait, and determining whether the patient has difficulty getting on the exam table can provide valuable information, which compensates for the small amount of extra time spent to gather these data. These functional assessments can easily be incorporated into clinical trials and have repeatedly been found to be highly predictive of various outcomes.^14^

The third missed opportunity is the chance to robustly analyze and report clinical trial data for the benefit of geriatric oncology research.^15,16^ This is particularly egregious, as the information exists—only forethought and a few simple steps are needed to extricate it. I commented on this in 2012, in response to the publication of a clinical trial in which older patients were a significant part of the study but no ­age-related data were reported (median age 58; range to 82 years).^15^ No age-related analysis was reported for efficacy or toxicity, and age distribution was not reported. Yet the drugs used in this trial included cisplatin and bevacizumab, which have increased toxicity in older patients. Since we accept the fact that clinical trial patients do not necessarily represent the patients seen in practice, then the older patients in this study were likely particularly fit to tolerate these medications. Where does this leave the clinician seeing a patient in routine practice who may want to use this regimen? Which patients should receive this regimen, according to which schedule, at what dose, and with what dose modification? None of this information was provided.

This study was not unique; many studies will report only a median age and range. To report results in this inadequate manner is a disservice to both the participants and the patients who will be treated based on the study results. A review of clinical trial publications confirmed the inadequacy of the reporting of data in older patients, despite the fact that an analysis of results by age should be simple from a statistical perspective and would not add to the cost of the trial.^16^ Such analyses would enhance the results and make them more useful to clinicians.

How can this be remedied? In order to advance geriatric oncology research, the necessary data needs to be collected, analyzed, and reported through appropriate representation of older patients, collection of essential and granular information, and thorough analysis and communication of results. Clinical trial design needs to include geriatric baseline parameters, according to the recommendations in the ASCO guidelines, and CTEP has also modified its template to include these items.^17^ Those responsible for the review of studies, ie, institutional review boards, the National Cancer Institute, and others, should require that these data be obtained and reported in the appropriate studies. In my opinion, one step that would be a game-changer would be the implementation of new standards by journal editors and reviewers. If one of the requirements for publication was age-appropriate data and analysis, investigators would be incentivized to incorporate these items into the design of trials. Journals require that studies conform to ethical standards. I submit that the ethical conduct of clinical trials includes the collection of all necessary data and the complete use of the data obtained. The readers need to demand this.

It is encouraging to see that the FDA has just released guidance for the inclusion of older patients in clinical trials.^18^ It is critical that the implementation of these recommendations be monitored to ensure compliance. Investigator-initiated and cooperative group trials also need to heed these recommendations.

I refer back to the earlier commentary I wrote for *The ASCO Post*, “Geriatric Assessment: What Are You Waiting For?”^19^ These simple and low-cost interventions will yield great dividends for the care of older patients.

## About the Author

Dr. Lichtman was an Attending Physician in the Department of Medicine, Memorial Sloan Kettering Cancer Center, and Professor of Medicine, Weill Cornell Medical College (retired). He is Past President of the International Society of Geriatric Oncology (2016-2018).
